# Delivering prehabilitation in cancer surgery: a service evaluation at Nottingham University Hospitals

**DOI:** 10.1007/s00520-026-11113-y

**Published:** 2026-09-15

**Authors:** Abdulrahman Alharbi, Ayleen Haywood, Dominic O’Connor

**Affiliations:** 1 King Abdullah Medical City, Makkah Al Mukarramah, Saudi Arabia; 2 School of Health Sciences, The University of Nottingham, Nottingham, England UK; 3 Nottingham University Hospitals, National Health Service, England, UK

**Keywords:** Prehabilitation, Cancer, Surgery, Service evaluation

## Abstract

**Purpose:**

To evaluate outcomes of a multimodal prehabilitation service for cancer surgery patients delivered through three pathways (Specialised, Targeted, Universal) within routine NHS care at Nottingham University Hospitals NHS Trust.

**Methods:**

This was a retrospective observational service evaluation of routine care data collected between 2022 and 2025. Of 1961 referred patients, 1720 were analysed: Specialised (*n* = 329), Targeted (*n* = 943), and Universal (*n* = 448). Multimodal prehabilitation included exercise, nutrition, and psychological support, stratified by risk. Functional capacity was assessed using the Incremental Shuttle Walk Test (ISWT), 60-s Sit-to-Stand (STS), and grip strength. Psychological well-being was measured using the Generalised Anxiety Disorder-7 (GAD-7), and Patient Health Questionnaire-9 (PHQ-9). Physical activity behaviour was also recorded.

**Results:**

All pathways showed significant within-group improvements. ISWT increased by 57 m (*p* < 0.001, *d* = 0.6) and STS by 6 repetitions (*p* < 0.001, *d* = 0.9). Anxiety (Δ –1.9) and depression (Δ –2.0) scores decreased (both *p* < 0.001, *d* ≈ –0.5). Weekly physical activity rose by 142 min (*d* = 1.07), and strength sessions increased by 2.4 per week (*d* = 1.1). Between-group differences were limited: PHQ-9 improved more in Specialised versus Targeted, and strength sessions increased more in Universal versus Targeted.

**Conclusion:**

A tiered, multimodal prehabilitation service integrated into cancer pathways produced meaningful functional, psychological, and behavioural benefits, supporting broader implementation and improved patient access.

**Supplementary information:**

The online version contains supplementary material available at https://doi.org/10.1007/s00520-026-11113-y.

## Introduction

Surgery remains central to cancer care, with 45 million procedures expected by 2030 annually worldwide [1]. Despite advancements, up to 40% of surgical patients develop complications such as blood clots or infections, leading to increased healthcare costs, prolonged hospital stays, delayed adjuvant therapy, and reduced long-term survival [2–4]. Surgical risk comes from a variety of factors including tumour biology, comorbidity, frailty, mental health, neoadjuvant therapy, and preoperative fitness [5–7]. Recognising that some of these are modifiable highlights the role of interventions such as prehabilitation to enhance surgical readiness and recovery.

Prehabilitation aims to optimise patients physical and psychosocial function before surgery to improve postoperative recovery [8, 9]. Typically delivered as a multimodal intervention combining exercise, nutritional, and psychological support [10], its format varies across settings. Hospital-based programmes with specialist supervision have been shown to improve aerobic capacity and reduce complications [11, 12]. Community-based programmes use local facilities to widen access and support lasting behaviour change. The Greater Manchester (GM) Prehab4Cancer (P4C) programme, for example achieved 50-m gains in the Incremental Shuttle Walk Test (ISWT) [13, 14]. More recently, Tele-prehabilitation further expands reach, enabling home-based participation with remote monitoring demonstrating high adherence, functional benefits, and strong patient acceptance [15–19].

National guidance now recommends a tiered risk-stratification approach. This includes the Universal–Targeted–Specialised model endorsed by Macmillan Cancer Support, which scales support by functional rather than chronological age [20, 21]. Within this framework, growing evidence suggests prehabilitation may reduce postoperative morbidity [22–29]. However, heterogeneity, small samples, and variable adherence limit certainty and generalisability [10, 30]. Studies suggest that benefits can begin to appear within two weeks of intervention [31, 32]. This highlights the potential for even brief intervention to add value. However, barriers to implementation remain common. These include short preoperative windows, variable clinician engagement, and patient factors such as travel demand or low motivation [33, 34]. Programmes appear most effective when multimodal, behaviourally supported, and flexibly delivered to accommodate patient needs [35, 36].

Hospital length of stay (LoS) remains a key indicator of recovery. However, evidence of prehabilitation’s impact are mixed. Small unimodal trials are often underpowered [37–39]. Systematic reviews suggest that multimodal programmes may reduce LoS by about two days. This is more pronounced when combined with nutrition and/or psychological components and aligned with Enhanced Recovery After Surgery pathways [40–43]. Economic evaluations are similarly encouraging. Multiple evaluations report per-patient cost savings, and a recent review concluded that prehabilitation is cost-effective in a majority of studies, albeit with variable quality [44–47]. To strengthen the evidence base, more standardised evaluation in routine clinical practice is needed.

Within this context, Nottingham University Hospitals NHS Trust established a multimodal prehabilitation service across Specialised, Targeted, and Universal pathways to align with national guidelines. The NUH Prehabilitation Service combines exercise, nutritional support and psychological preparation to optimise patients before cancer surgery. A service-evaluation approach is proportionate for assessing real-world impact and informing quality improvement [48–50]. This evaluation examines changes in functional capacity, psychological well-being, and physical activity across the three pathways. The aim of this study is to guide local development and contribute to the wider evidence base on prehabilitation models.

## Methodology

This evaluation was reported in accordance with the Standards for Quality Improvement Reporting Excellence (SQUIRE 2.0) framework, which supports transparent, contextual reporting of healthcare service initiatives [51]. Methods are presented under the SQUIRE domains of Context, Intervention, Study of the Intervention, Measures, Analysis, and Ethical Considerations.

### Context

This service evaluation examined the cancer prehabilitation service delivered at Nottingham University Hospitals NHS Trust (NUH) between April 2022 and May 2025. The NUH PrehabilitationService is structured into three delivery pathways that align with Macmillan prehabilitation guidelines [20]. Patients were allocated to pathways using a clinical risk stratification approach [52] based on factors including frailty, functional capacity, comorbidity, and treatment complexity. Higher-risk patients were assigned to more intensive pathways (full stratification criteria detailed in Supplementary File A).

### Staffing

The Prehabilitation Team included three whole-time-equivalent clinical staff (physiotherapist and exercise professional/physiologist), a patient pathway coordinator, a pre-operative and prehabilitation matron, and supporting consultant anaesthetists. The wider team also involved the community exercise provider *A Better Life (ABL)*, social prescriber/link worker support through *Self-Help UK*, and psychology input from East Midlands Cancer Alliance Centre for Psychosocial Health.

### Referral pathway and eligibility

Patients were eligible for prehabilitation if they were adults (18 +) on a confirmed cancer-surgery pathway and had at least three weeks before their planned theatre date. Referrals were encouraged as early as possible, ideally before the first cycle of neoadjuvant therapy, but the programme continued to accept referrals at any stage before surgery, including during or after completion of neoadjuvant treatment. Surgical targets included major resections across multiple specialties.

- Colorectal (e.g. major ano-rectal or colonic resections)
- Hepato-pancreato-biliary (primary or metastatic liver resection, Whipple’s procedure)
- Gynaecological oncology (ultra-radical hysterectomy, posterior pelvic exenteration, midline laparotomy)
- Upper GI (gastrectomy, oesophagectomy)
- Urology (cystectomy, open nephrectomy)
- Head and neck (maxillofacial cancer resection with free-flap reconstruction)
- Thoracic surgery for cancer (lung or mediastinal masses) in Nottinghamshire residents
- Germ-cell tumours with surgical targets such as retro-peritoneal lymph-node dissection

Exclusion criteria: active infection (e.g. COVID-19), severe cognitive impairment that prevented participation, time to surgery under 3 weeks, or patients on oncology treatment with no surgical procedure planned.

### Intervention

The NUH Prehabilitation Service delivers three pathways, each initiated immediately after risk stratification:

1. Specialised (high-risk patients): Patients attend supervised exercise with the NUH Prehab team up to three times a week. Sessions are delivered on a one-to-one or two-to-one basis, allowing close monitoring and individualised progression.
2. Targeted (lower-risk patients): Patients participate in community-based group classes, up to three times a week, that combine modified high-intensity interval training (HIIT) with strength exercises. Delivery is led by the external provider, ABL, under NUH oversight.
3. Universal (“Arms-length” offer): Independently active or out-of-area patients receive an individualised exercise prescription to be done at home and maintain weekly check-ins with the prehabilitation team for education and advice.

### Design

This retrospective service evaluation analysed routinely collected data from the NUH Prehabilitation Service. The evaluation used existing clinical data and involved no experimental intervention or deviation from standard care. All participants received care according to standard triage protocols.

### Eligibility for this service evaluation

#### Inclusion criteria

- Referred to the NUH Prehabilitation Service between April 2022 and May 2025.
- Aged ≥ 18 years
- Allocated to one of the three delivery pathways
- Had both a baseline and a matched post-intervention value for the outcome under analysis

#### Exclusion criteria

- Did not start the prescribed intervention
- Missing either the baseline or the post-intervention value for the outcome under analysis
- Time in the service > 365 days, a threshold set by the service provider as an outlier rule

### Participant flow

An anonymised Excel dataset containing 1962 referral records, prepared and supplied by the NUH Prehabilitation Team, was screened in three stages. First, the entire referral dataset was reviewed for data integrity and completeness. Second, pre-specified cleaning rules were applied to remove ineligible or incomplete records. Third, the remaining cases formed the analysis set for all subsequent statistical tests. Sample sizes, therefore, vary by outcome measure and are reported in the “Results” section.

### Data source and management

This service evaluation used anonymised secondary data provided by the Prehabilitation Team at NUH NHS Trust. The extract contained every patient referred to the NUH Prehabilitation Service as part of the routine pre-operative pathway for elective cancer surgery. The data were collected for clinical monitoring, not for research, and were received anonymised. No identifiable patient information was included.

### Contents of the dataset

- Demographics: age, gender, ethnicity, BMI
- Clinical variables: surgical speciality, frailty score, receipt of neoadjuvant therapy
- Outcome measures: baseline and post-intervention scores for each measure used in the evaluation
- Engagement indicators: number of sessions and total time in service. Engagement data were recorded in total and could not be broken down into specific contact types (e.g. assessments, exercise sessions, or telephone calls).

### Outcome measures

The Prehabilitation Team collected the following outcomes as part of routine monitoring (physical function, psychological status, and service engagement); see supplementary file B for date collection time-points by the prehabilitation team:

- Baseline-only descriptors. BMI; Clinical Frailty Scale.

### Functional outcomes

- ISWT: ISWT is an externally paced, maximal 20-min protocol that increases walking speed with each audio-cued level until the participant can no longer maintain pace and is a validated test of cardiopulmonary exercise capacity in different populations [53, 54].
- 60-Second Sit-to-Stand: Offers a quick, space-efficient measure of lower-limb strength, endurance, and exercise capacity [55, 56]. The test shows strong convergent and known-groups validity and sufficient test–retest reliability [56].
- Grip Strength (left and right). Maximal voluntary force (kg) recorded with a hand-held dynamometer; higher values denote stronger grip [57]. Hand-grip strength is a well-validated measure of overall health and frailty in older adults [57].

### Psychological outcomes

- PHQ-9: A self-report instrument for screening, diagnosing, monitoring and grading depression severity [58].
- GAD-7: A brief self-report measure for identifying and quantifying generalised anxiety disorder [59].

### Physical activity outcomes

- Self-reported physical-activity minutes per week. Participants estimate moderate-to-vigorous activity; greater minutes signify higher activity.
- Strength-session. Self-reported count of the prescribed strength-training sessions, or equivalent strength exercises, completed during the programme.

### Analysis

All statistical analysis were conducted in Microsoft Excel (Office 365) using the Real Statistics add-in. The significance threshold was set at p < 0.05 (two-tailed).

### Within-group change

For every outcome collected at both baseline and service completion, a paired t-test compared pre- and post-means within each group (Specialised, Targeted, Universal).

### Between-group comparison

Change scores (Post–Pre) were compared across the three groups with a one-way ANOVA. When the ANOVA F-ratio reached significance (two-tailed p < 0.05), the Real Statistics add-in Tukey’s Honestly Significant Difference (HSD) post-hoc test was applied to identify specific pairwise group differences. Between-group comparisons were conducted without adjustment for baseline differences and are therefore considered exploratory.

### Effect sizes

- Cohen’s d (within-group) was calculated as the mean change divided by the pooled standard deviation of the two time-points.
- Eta-squared (η^2^) was taken directly from the ANOVA output to estimate the proportion of variance explained by the programme group.

### Missing data

Missing data were handled per outcome using a complete case approach; participants were included in each analysis only if they had complete (pre- and post-) data for that outcome. They were not excluded from the overall dataset if they were missing data for other variables. This approach assumes the data are missing completely at random. If this assumption is not met the results may be subject to bias. Differential loss of participants may introduce attrition bias, and the magnitude of observed effects may be overestimated or underestimated.

## Results

### Participant characteristics

**Fig. 1:**
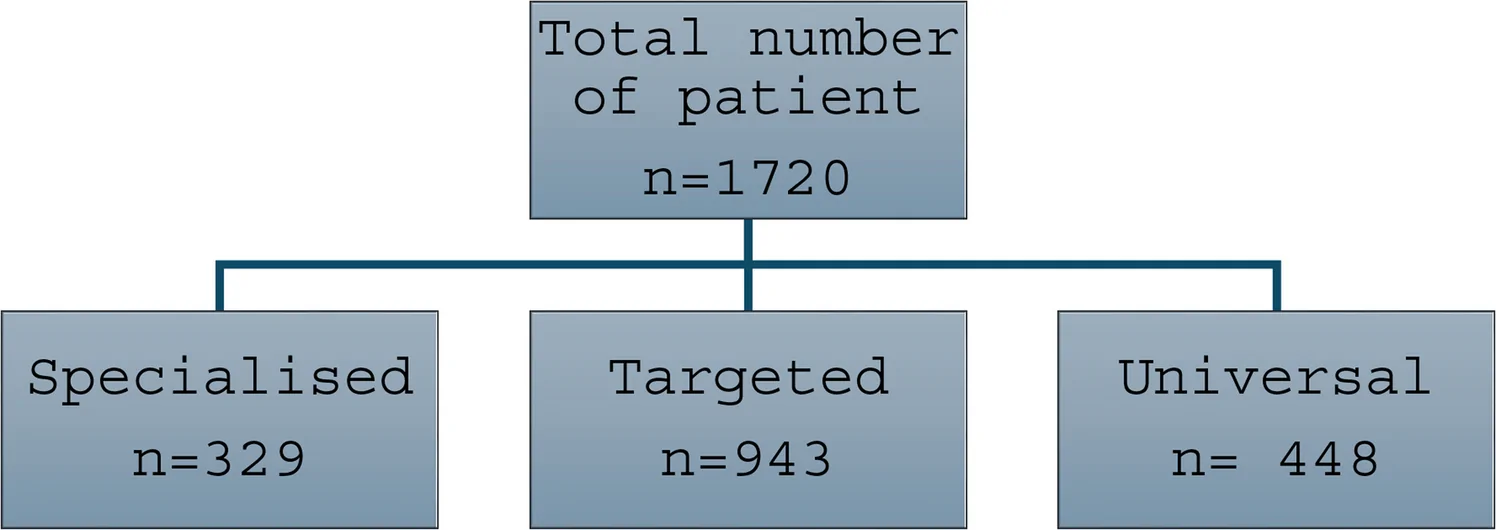
The distribution of participants across programme pathways

Of the 1961 patients referred, 1720 were included in the analysis (241 excluded due to missing data). The Prehabilitation Team allocated patients into three pathways: Targeted (*n* = 943, 54.9%), Universal (*n* = 448, 26.0%), and Specialised (*n* = 329, 19.1%) (Fig. 1).

The cohort had a median age of 67 years (IQR 58–74) and included more females (52.5%) than males (47.5%). Nearly half (49.4%) were classified as obese, with only 15.6% in the normal BMI range. Frailty data (available for 865 patients) showed a mean score of 2.53 (SD 1.16). Neoadjuvant treatment had been received by 16% of patients (Table 1).

**Table 1:** Patient characteristics (*n* = 1719). Speciality distribution: colorectal surgery 663 (38.5%), gynaecological oncology 349 (20.3%), hepatobiliary and pancreatic surgery 191 (11.1%), upper gastrointestinal 168 (9.8%), urology 161 (9.4%), other 187 (10.9%)

Age (median [IQR])	67 years (IQR 58–74)
Gender	
Male	817 (47.5%)
Female	903 (52.5%)
Ethnicity	
White British	1119 (65%)
Unknown	322 (18.7%)
Declined to provide their ethnicity	147 (8.6%)
Other	132 (7.7%)
BMI	
Obese (≥ 27.5)	849 (49.36%)
Overweight (23–27.5)	550 (32%)
Normal	268 (15.6%)
Underweight	41 (2.4%)
Frailty (*n* = 865) (mean; SD)	2.53 (1.16)
Neoadjuvant treatment	
Yes	276 (16%)
No	1168 (68%)
Missing data	276 (16%)

The specialised group had a slightly higher Frailty score (3.4, SD = 1.1) and BMI (29.6, SD = 8.66) compared to the other groups (Table 2). Given baseline differences between groups, between-group comparisons are presented as exploratory.

**Table 2:** Baseline characteristics of participants by group

Group	Age mean (SD)	BMI mean (SD)	Frailty mean (SD)
Specialised	66.0 (11.9)	29.6 (8.7)	3.4 (1.1)
Targeted	65.0 (11.7)	28.2 (5.5)	2.2 (1.0)
Universal	65.5 (11.5)	27.8 (6.0)	2.4 (1.2)

### Service engagement

A total of 8250 service sessions were delivered to 1720 patients, with the service structured around three sessions per week. Attendance varied by pathway: Specialised (10.97 sessions), Universal (4.65 sessions), and Targeted (2.90 sessions), for an overall average of 4.9 sessions per patient.

Time in the service was available for 1570 patients (91.5%), with data missing for 145 patients (8.5%). Duration ranged from 0.1 to 363.8 days, with a median of 33.4 days (IQR 16.8–66.0), equivalent to 2.5–9.5 weeks of engagement. The distribution was right-skewed due to a small number of long-duration cases. Ninety-one patients had 10 days or fewer in the service, of whom only one had post-intervention data.

Documented reasons for non-completion were available for 36 patients: short timeframes before surgery (*n* = 14), non-attendance at assessment (*n* = 3), and discharge after assessment (*n* = 19), often due to preference or low perceived need. A further 34 engaged for < 2 weeks, with 15 still completing follow-up. Reported barriers included travel distance, competing appointments, and perceptions of adequate baseline fitness.

### Outcome measures

Significant improvements were observed across all domains (Table 3). Functional capacity increased, with the largest gain seen in the ISWT (+ 57 m). Psychological outcomes (anxiety and depression) improved, and physical activity more than doubled (+ 142 min per week), including a marked rise in weekly strength sessions (+ 2.4).

**Table 3:** Outcome measures

Outcome measure	Participants (n)	Pre mean (SD)	Post mean (SD)	Mean change (Δ)	Cohen’s *d*	*p* value
ISWT (meters)	459	346 (181.4)	403.3 (184.7)	+ 57.2	0.6	< 0.001
Sit-to-STand (Reps)	632	23.2 (8.7)	29.2 (9.8)	+ 6	0.9	< 0.001
Grip strength left (kg)	495	25.7 (10.3)	26.7 (10.2)	+ 1	0.3	< 0.001
Grip strength right (kg)	495	26.8 (10.8)	28 (10.7)	+ 1.2	0.3	< 0.001
GAD-7 (anxiety score)	831	6 (5.66)	4 (4.55)	− 2	− 0.48	< 0.001
PHQ-9 (depression score)	824	5.71 (5.68)	3.68 (4.54)	− 2.02	− 0.51	< 0.001
Physical activity (min/week)	793	111 (215)	253 (215)	+ 142	1.07	< 0.001
Strength sessions per week	784	0.61 (1.5)	3 (2.13)	+ 2.43	1.1	< 0.001

### Post-operative questionnaire

The Prehabilitation Team provided post-operative questionnaires for a subgroup of patients; however, these were not included in the structured outcomes dataset. A total of 662 patients completed the 3-month post-operative questionnaire which assessed perceived benefit and behavioural change following completion of prehabilitation (see supplementary file D), reflecting a 72% response rate between 2022 and 2025. Responses suggested that 83.3% of patients agreed or strongly agreed that prehabilitation helped them prepare for surgery, and 61.1% felt it supported their recovery. However, only 31.2% reported being more physically active post-operatively, while 38.1% felt less active. Notably, 34.3% of respondents were still undergoing cancer treatment at the time of response.

### Analysis of outcome improvements by group

PHQ-9 scores decreased, and weekly strength sessions increased in all groups. On both outcomes, Specialised and Universal improved more than Targeted, with no difference between Specialised and Universal. For anxiety, aerobic capacity, sit-to-stand, grip strength, and overall physical activity, there were no between-group differences. See the Supplementary files C.

## Discussion

To our knowledge, this represents the largest service evaluation of a multimodal, tiered cancer prehabilitation service conducted to date, spanning a 38-month service delivery period (April 2022–May 2025) and encompassing 1720 patients across seven surgical specialities. This equates to an average of 52 eligible referrals per month, distributed across three care pathways: Specialised (*n* = 329), Targeted (*n* = 943), and Universal (*n* = 448). These participation levels indicate strong engagement from referring clinical teams.

The median time in the service was 33.4 days (IQR 16.8–66.0), aligning with evidence showing that, while benefits can emerge within two weeks [32], programmes lasting more than three weeks are more likely to improve postoperative complications [60]. The wide IQR likely reflects heterogeneity in cancer type, treatment pathway, and individual patient need. The NUH Prehabilitation Service uses a wide range of outcome measures to address physical, psychological and behavioural domains, allowing patients to be stratified according to their needs and abilities. This approach enables early identification of those most likely to benefit, supports enrolment into the appropriate level of intervention, and when combined with adequate adherence and sufficient duration, maximises the potential to enhance health status before surgery [30]. However, the duration of prehabilitation and proximity to surgery likely varied across tumour type and pathways. This may have influenced the magnitude of observed changes and should be considered when interpreting outcomes.

Attendance across the service highlights both engagement and data limitations. The Specialised pathway mostly met its intended exercise dose, while data for Targeted were unavailable. In the Universal pathway, telephone contacts were recorded as clinic contacts rather than exercise sessions, which may overestimate support. These patterns reflect common barriers such as hospital appointments, caring responsibilities, and transport issues [36, 61, 62]. A key limitation is that contacts could not be disaggregated into assessments, reassessments, exercise sessions, and telephone calls, which may affect interpretation of engagement. Future evaluations should address this to provide a clearer picture of adherence and participation across pathways.

Across all pathways, patients demonstrated statistically significant improvements in all outcome measures. The Specialised pathway, which received intensive support, showed greater improvements in PHQ-9 scores compared with both the Targeted and Universal pathways, and a higher number of completed strength-training sessions per week compared with the Targeted pathway only. For other outcomes (e.g. ISWT, GAD-7, grip strength, physical activity), between-group differences were not statistically significant, indicating that the tiered model may be effective in improving patient outcomes across all starting levels.

### Psychological outcome measures

PHQ-9 scores decreased significantly across all pathways, with moderate effect sizes (Cohen’s *d* = 0.45–0.51), aligning with evidence of prehabilitation reducing psychological distress in surgical cancer populations [63]. The overall mean change (− 2.02) met the minimal clinically important difference (MCID) for the PHQ-9 established as approximately 2 points in UK primary care cohorts [64]. This suggests that the NUH Prehabilitation Service achieved a clinically meaningful benefit based on the MCID. Between-group analysis showed a statistically significant advantage for the Specialised group compared to the Targeted group (*p* = 0.0019, *d* = 0.30). This is supported by broader findings showing that interventions with more structured, individualised psychological support, rather than generic, produce greater improvements in psychological wellbeing before surgery [65, 66]. As such, there may be added value from more tailored support for psychological well-being during prehabilitation. However, it remains unclear whether the observed improvements persist after surgery, as most data, including the present cohort, were limited to preoperative reassessment.

In contrast, no statistically significant between-group differences were observed in GAD-7 anxiety scores, suggesting that general engagement with prehabilitation may be sufficient to improve anxiety regardless of delivery pathway. In the literature, studies in oncology populations suggest that the conventional GAD-7 threshold of 10 may underestimate anxiety prevalence in cancer populations, with enhanced sensitivity observed at lower cut-offs of approximately 7–8 [67]. This supports a more cautious interpretation of absolute scores in surgical oncology, particularly where symptom presentations may be subtle. In this cohort, baseline scores averaged 6, below the adjusted threshold, and reduced to 4 post-interventions, a statistically significant reduction (*p* < 0.001) but below the established MCID of 4 points [68]. This suggests that the change may not be clinically meaningful, particularly given the low baseline anxiety levels. Taken together, these findings suggest that while the NUH Prehabilitation Service delivered improvements in depression (PHQ-9), its effect on anxiety (GAD-7) may not be clinically meaningful, highlighting the need for more tailored approaches to address anxiety within prehabilitation.

### Functional and physical capacity

Objective assessments of functional and physical capacity provided evidence of positive change during the evaluation period. Performance on the 60s-STS improved across the cohort, with mean gains of 6 repetitions exceeding the MCID of 2–3 repetitions [69, 70]. Comparable gains were observed in the P4C programme (+ 4.8 repetitions, 95% CI 3.5–6.0) [13], providing supportive evidence of consistency with other NHS prehabilitation cohorts. However, post-intervention means remained below normative values reported in healthy adults of comparable age [71], which may reflect not only the cumulative impact of cancer and comorbidities but also the deconditioning effects of neoadjuvant treatment. Similar benefits have been reported in cancer prehabilitation cohorts using the 30s-STS, a measure with stronger validation and feasibility in surgical oncology [72]. The absence of between-group differences suggests that functional gains may be achievable across all delivery models when engagement is maintained.

The use of the 60s-STS warrants caution. Although capable of detecting endurance-related change, it is less validated in cancer surgery than the 30s-STS, which benefits from more robust normative data and wider application [72–74]. The longer duration also risks introducing fatigue, degraded form, or test termination in frailer patients, reducing comparability and potentially creating a floor effect. Evidence from chronic disease populations shows that a notable minority cannot complete the 60s-STS but manage shorter-duration variants [55, 75]. This highlights the importance of selecting measures that balance sensitivity to change with feasibility and safety in high-risk cohorts.

For the ISWT, clinically meaningful improvements were observed. Minimal important differences for rehabilitation in long-term conditions are typically 35–53 m [76, 77], and all service pathways exceeded this range, with gains of 43 m (Universal), 56 m (Targeted), and 64 m (Specialised). The Specialised pathway exceeded the upper MID threshold, suggesting that more intensive provision may yield additional benefits. However, these patients were generally frailer and had higher comorbidity, meaning even modest exercise interventions could produce improvements in balance, confidence, and general mobility, alongside fitness and functional capacity. This raises the question of whether the observed gains reflect intervention intensity or simply the effect of initiating any structured activity in a deconditioned population. These findings align with the P4C programme, which reported mean ISWT improvements of approximately 50 m [13]. Collectively, these findings suggest that prehabilitation may deliver clinically meaningful gains in functional capacity, with magnitude scaling to programme intensity.

Physical activity has been widely shown in the literature to benefit people with cancer [78, 79], including enhancements in mental health, quality of life, and reductions in cancer recurrence and mortality [80]. In this evaluation, physical activity data were available for 793 participants. The proportion reporting any weekly activity increased from 54% at baseline to 100% post-intervention. This suggests a meaningful behavioural shift; however, such findings must be interpreted cautiously given the reliance on self-reported measures, which are prone to recall bias and social desirability effects [81]. The service likely enhanced capability by increasing patients’ knowledge of appropriate preoperative exercise (psychological capability) and by improving their physical skills and confidence through tailored prescription and supervised practice (physical capability). It created opportunity via structured sessions and accessible support and strengthened motivation through regular assessment and feedback. Similar behavioural science applications in prehabilitation have demonstrated effectiveness in increasing preoperative activity levels [82, 83].

### Strengths

This evaluation of 1720 patients across Specialised, Targeted and Universal pathways is one of the largest service-level analyses of cancer prehabilitation. A key strength is that it reflects delivery within routine clinical care, rather than a research driven intervention. This enhances real-world relevance and ecological validity. The inclusion of a large, diverse cohort spanning seven surgical specialities supports the generalisability of findings across a broad cancer population.

Strengths also include the wide range of outcomes assessed, the use of pre and post comparisons with between-group testing, and the inclusion of Cohen’s d effect sizes to aid clinical interpretation. Finally, the scale and tiered structure of the service provide valuable insights for NHS service planning, scalability, and commissioning of prehabilitation services.

### Limitations

This service evaluation has several limitations. Analyses were conducted using a complete case approach, where only participants with both pre- and post-intervention scores were included. This approach assumes that data are missing completely at random. If this assumption is not met, the findings may be subject to attrition bias as participants with incomplete data may differ systematically from those included in the analysis. This may have inflated estimates of effectiveness or otherwise biased effect sizes. A similar issue was reported in GM P4C, where less than half of baseline data had paired results [13], highlighting how missingness may affect interpretation across services.

The observational design and lack of randomisation also limit causal inference. In addition, between group comparisons were conducted without adjustment for baseline differences, and should therefore be interpreted as exploratory, as observed differences may reflect underlying patient characteristics rather than service effects.

Interpretation of physical activity outcomes is further limited by the use of self-reported physical activity measures, which are prone to recall and social desirability bias and may have resulted in overestimation of activity levels and inflation of observed effect sizes.

Selection bias may be present upstream of service enrolment. Referral into the NUH Prehabilitation Service was dependent on clinician discretion, tumour type, and service capacity. This may not reflect the full eligible patient population. Furthermore, patients allocated to lower-intensity pathways may represent a relatively healthier or more motivated subgroup, which could contribute to observed differences between pathways independent of intervention effects.

Interpretation of clinical significance is further constrained by the absence of universally accepted MCID thresholds for cancer populations, given the variability in symptoms, treatment stage, and baseline function [84]. The absence of postoperative clinical outcomes (e.g. length of stay, complication rates) also limits the ability to assess whether observed preoperative improvements translated into meaningful downstream surgical benefits.

Data on reasons for non-engagement were incomplete, though common barriers included short pre-surgery windows, travel difficulties, and perceptions of being either already fit or too overwhelmed. While delivered in line with Macmillan’s Principles and Guidance, these contextual issues limit generalisability.

### Implications for clinical practice

The findings support the use of a stratified, tiered approach to prehabilitation, whereby patients are allocated to intervention intensity based on clinical need. Improvements observed across all pathways suggest that meaningful benefit can be achieved even within lower-intensity pathways. Greater gains in psychological outcomes within the Specialised pathway highlight the added value of more tailored and supervised support for higher-risk patients. These results suggest that Universal or arm’s-length pathways may provide a viable and scalable option for supporting lower-risk or more independent patients, particularly in the context of service capacity constraints. More broadly, this demonstrates applicability within publicly funded healthcare systems such as the NHS, where multidisciplinary allied health services are embedded. However, transferability may be more limited in settings without comparable infrastructure.

### Recommendations for service improvement

1. Include hospital length of stay as an outcome measure:

LoS is a key prehabilitation outcome linked to recovery, complications, and cost savings [42, 43], Its absence limits comparability and weakens the health-economic case. Routine LoS collection would strengthen benchmarking and investment arguments.

2. Mitigate barriers to engagement:

Travel, scheduling, and perceptions of adequate fitness were frequent barriers. Adapting targeted/universal pathways with hybrid delivery, digital follow-up, and clearer clinician led communication regarding prehabilitation benefits [85] may improve accessibility, adherence, and uptake.

## Conclusion

This evaluation showed that a multimodal cancer prehabilitation service, aligned with Macmillan guidance, was linked to improvements in mood, strength, and functional capacity, supporting patients’ surgical preparation and recovery. Engagement challenges, including short pre-surgery windows and logistical barriers, highlight the need for stronger referral pathways and flexible delivery models. More structured post-operative follow-up and systematic data capture would further enhance service development. Overall, the findings support integrating prehabilitation into routine cancer care and underline the need for ongoing evaluation to maximise accessibility and impact.

## Supplementary information

Below is the link to the electronic supplementary material.

Supplementary file1 (DOCX 23 kb)

## Data Availability

The data used in this service evaluation were collected routinely as part of clinical service monitoring by the Nottingham University Hospitals NHS Trust Prehabilitation Service and were provided to the authors in anonymised form. The data are not publicly available because they are owned by Nottingham University Hospitals NHS Trust and are subject to governance and confidentiality requirements.
