# Supplementary material for: Delivering prehabilitation in cancer surgery: a service evaluation at Nottingham University Hospitals
Source: Support Care Cancer. 2026 Sep 15;34(10):973. doi: 10.1007/s00520-026-11113-y (PMC13574862; doi:10.1007/s00520-026-11113-y)
Supplement: Supplementary file 1 [file 520_2026_11113_MOESM1_ESM.docx]

Supplementary file A: local stratification criteria aligned with Macmillan guidelines;

***Specialised group (high-risk patients)***

*General Health*

- Eastern Cooperative Oncology Group (ECOG) performance status > 2
- Presence of multiple co-morbidities
- Extensive cardiac history or recent myocardial infarction/cardiac surgery (< 6 months ago), angina on exercise
- Extensive neurological history or recent neurological event
- Body mass index (BMI) over 40
- Poorly controlled diabetes
- Severe cachexia/sarcopenia
- Severe anxiety preventing participation in group activity
- Cognitive impairment

*Cancer/Treatment*

- Haemoglobin < 100; should also be referred for iron studies/anaemia support if less than 130
- Platelets < 75.
- Presence of bone metastasis and cleared to exercise by oncologist.

***Outcome Measures***

- ISWT: achieves less than 250 m, SpO2 drops by more than 4%, stops due to chest pain
- High resting heart rate (> 100) and/or high Blood pressure (> 190 systolic or > 115 diastolic)

***Targeted Exercise Intervention (lower-risk patients)***

Patient needs supervised input but is deemed safe to exercise in a community-based programme with none of the points noted for the Specialised group.

***Universal Exercise Intervention (‘Arms-length’ offer)***

- Patient greatly exceeds recommended physical activity guidelines
- No significant past medical history
- Out-of-area patient/declines targeted intervention

supplementary file B : Date collection time-points by the prehabilitation team

| Timepoint | Measures/Assessments |
| --- | --- |
| Pre-intervention | - Demographic information - BMI - ISWT - Sixty-second sit-to-stand - Grip strength - GAD-7 - PHQ-9 - Self-reported physical activity - Self-reported strength session - Clinical Frailty Scale |
| Post-intervention | Reassessment of all previous measures except:   - Demographic information - BMI - Clinical Frailty Scale |

Supplementary file C-1 : Changes in PHQ-9 score by group

| Group | Pre Mean | Post Mean | Mean Change (Δ) | Paired t-test (p-value) | Cohen’s d (within) | Tukey Post-hoc Comparison |
| --- | --- | --- | --- | --- | --- | --- |
| Specialised | 8.20 | 5.29 | –2.91 | < 0.001 | 0.51 | vs Targeted (p = 0.0019, d = 0.30)  vs Universal (p = 0.144, d = 0.22) |
| Targeted | 4.97 | 3.25 | –1.72 | < 0.001 | 0.45 | vs Specialised (p = 0.0019, d = 0.30)  vs Universal (p = 0.0022, d = 0.33) |
| Universal | 5.32 | 3.27 | –2.05 | < 0.001 | 0.51 | All comparisons (p > 0.05) |

Supplementary file C-2 : Changes in strength session by group

| Group | Pre Mean | Post Mean | Mean Change (Δ) | Paired t-test (p-value) | Cohen’s d (within) | Tukey Post-hoc Comparison |
| --- | --- | --- | --- | --- | --- | --- |
| Specialised | 0.33 | 3.00 | +2.67 | < 0.001 | 0.66 | vs Targeted (p = 0.050, d = 0.21) |
| Targeted | 0.62 | 2.83 | +2.21 | < 0.001 | 0.61 | vs Universal (p = 0.002, d = 0.33) |
| Universal | 0.95 | 3.89 | +2.94 | < 0.001 | 0.64 | vs Targeted (p = 0.002, d = 0.33)  vs Specialised (ns) |

Supplementary file C-3 : GAD-7 – Between-Group Comparison (ANOVA)

| **Group** | **n** | **Mean Change (Δ)** |
| --- | --- | --- |
| Specialised | 177 | –2.11 |
| Targeted | 519 | –1.85 |
| Universal | 135 | –1.66 |
| ANOVA p-value | – | 0.573 |

Supplementary file C-4 :60-Sit-to-Stand – Between-Group Comparison (ANOVA)

| **Group** | **n** | **Mean Change (Δ) (repetitions)** |  |
| --- | --- | --- | --- |
| Specialised | 142 | 6.32 |  |
| Targeted | 410 | 5.93 |  |
| Universal | 80 | 6.04 |  |
| ANOVA Total | – | – | 0.834 |

Supplementary file C-5: Physical Activity – Between-Group Comparison (ANOVA)

| **Group** | **n** | **Mean Change (Δ) (Min per week)** |  |
| --- | --- | --- | --- |
| Specialised | 163 | 146.32 |  |
| Targeted | 507 | 145.95 |  |
| Universal | 123 | 122.66 |  |
| ANOVA Total | – | – | 0.200 |

Supplementary file C-6: Comparison Incremental Shuttle Walk Test (ISWT)

| **Group** | **Post Mean change (meter)** | **n** | **ANOVA (p-value)** |
| --- | --- | --- | --- |
| Specialised | 63.83 | 134 |  |
| Targeted | 55.78 | 289 |  |
| Universal | 43.33 | 36 |  |
| **ANOVA** | – | – | **0.465** |

Supplementary file C-7: Group Differences in Grip Strength Improvement

| **Grip Side** | **Group** | **Mean Change (Δ) (kg)** | **ANOVA p-value** |
| --- | --- | --- | --- |
| **Left** | Specialised | 1.32 | 0.416 |
|  | Targeted | 0.86 |  |
|  | Universal | 1.05 |  |
| **Right** | Specialised | 1.35 | 0.939 |
|  | Targeted | 1.28 |  |
|  | Universal | 1.51 |  |

Supplementary files D:Three-month Post-operative Service Questionnaire

**Questions and response options**

1. Prehab helped me to prepare for surgery
    - Strongly agree
    - Agree
    - Neither agree nor disagree
    - Disagree
    - Strongly disagree
    - Unsure
2. Prehab helped me to recover from surgery
    - Strongly agree
    - Agree
    - Neither agree nor disagree
    - Disagree
    - Strongly disagree
    - Unsure
3. Compared to before entering the prehab programme, how physically active are you now?
    - Less active than before prehab
    - The same as before prehab
    - Slightly more active than before prehab
    - Significantly more active than before prehab
    - Unsure
